# Clinical Outcomes After Catheter Ablation in Patients With Structural Heart Disease and Ventricular Fibrillation

**DOI:** 10.1111/jce.70488

**Published:** 2026-08-30

**Authors:** Kenji Hashimoto, Samual Turnbull, Saurabh Kumar

**Affiliations:** ^1^ Department of Cardiology, Westmead Hospital, Westmead Applied Research Centre University of Sydney Sydney Australia

**Keywords:** cardiomyopathy, catheter ablation, ventricular fibrillation

## Abstract

**Introduction:**

In patients with ischemic cardiomyopathy (ICM), recurrent VF may be treated with catheter ablation by targeting triggering premature ventricular complexes (PVCs) and/or ventricular scar homogenization. Data on optimal ablation strategies in patients with non‐ischemic cardiomyopathy (NICM) remain limited. This study aimed to compare ablation strategies and outcomes in patients with recurrent VF due to ICM versus NICM.

**Methods and Results:**

We retrospectively analyzed consecutive patients with structural heart disease and recurrent VF undergoing catheter ablation. Catheter ablation was performed, including targeting the triggering and/or clinically frequent PVC, targeting any inducible ventricular tachycardia, and/or scar homogenization. Procedural characteristics, arrhythmogenic substrate features, and post‐ablation clinical outcomes were compared between ICM and NICM groups. Forty‐five patients were included (17 ICM, 28 NICM; mean age 60 ± 15 years; 87% male). PVC ablation was performed in 42% of ICM and 39% of NICM patients, while scar homogenization was undertaken in 76% and 57%, respectively. Left ventricular scar burden, assessed by bipolar and unipolar voltage mapping, was significantly greater in the ICM, whereas the prevalence of abnormal Purkinje potentials was similar between groups. VF‐free survival after multiple procedures did not differ between groups, with estimated 1‐year rates of 87 ± 9% in ICM and 85 ± 8% in NICM (log‐rank *p* = 0.93).

**Conclusions:**

In patients with NICM and recurrent VF, catheter ablation targeting arrhythmogenic substrate—including unipolar low‐voltage regions—combined with PVC ablation yields outcomes comparable to those in ICM, supporting this approach as a reasonable treatment strategy.

## Graphical abstract

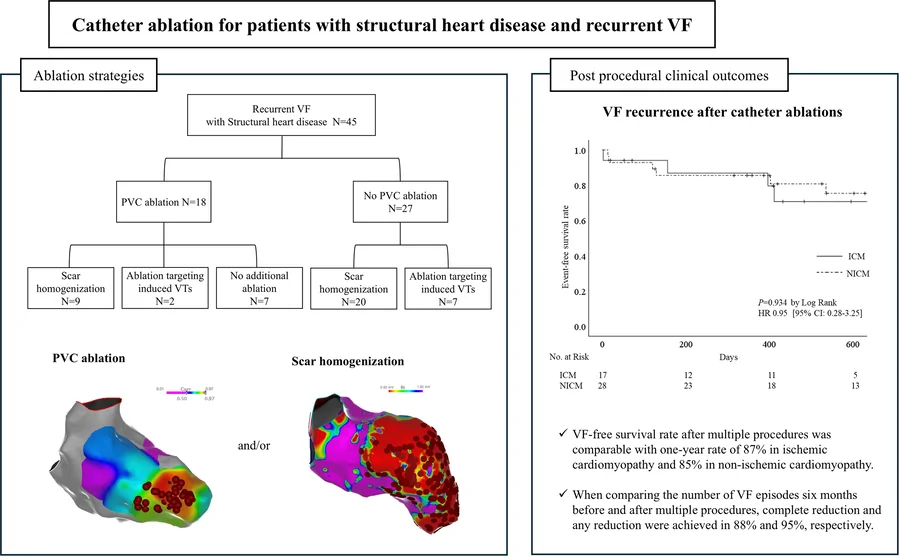

## 1 Introduction

Patients with structural heart disease (SHD) may experience recurrent ventricular fibrillation (VF), as well as monomorphic ventricular tachycardia (VT). Catheter ablation (CA) has been shown to be effective for reducing both VT and VF recurrences [1]. Multiple ablation strategies have been proposed to ablate VF. Previous reports have mainly focused on the CA of VF‐triggering premature ventricular complexes (PVC), but this approach may be hampered by PVC quiescence at the time of ablation [2]. An alternative strategy may involve the modification of arrhythmogenic substrate, which may alleviate future VF episodes, as seen in patients with Brugada syndrome, wherein elimination of epicardial right ventricular (RV) substrate reduces VF recurrence [3]. In ischemic cardiomyopathy (ICM), the Purkinje system is considered to play a key role in initiating and maintaining VF and could be targeted for ablation to achieve freedom from VF recurrence [4, 5]. In addition, scar homogenization in ICM has been reported to reduce VF burden [1]. More recently, targeting the Purkinje system and additional arrhythmogenic substrate modification in patients with idiopathic VF, non‐ischemic cardiomyopathy (NICM), and mixed cardiomyopathy has also been shown to reduce VF burden [6, 7].

Whilst the efficacy of triggering PVC ablation and/or substrate homogenization has been demonstrated, these reports predominantly included patients with ICM. Only a small number of NICM patients were included in prior studies; therefore, data on appropriate strategies and clinical outcomes for NICM patients are limited.

The aim of this study was (i) to describe the arrhythmogenic substrate, ablation strategies, and long‐term outcomes in patients undergoing CA for VF related to SHD; (ii) to compare substrate characteristics and clinical outcomes of ablation for VF in patients with NICM to those with ICM; and (iii) to compare the substrate of NICM patients with VF versus VT to investigate VF‐specific substrate.

## 2 Methods

Patients with SHD undergoing CA for ventricular arrhythmia (VA) between January 2019 and September 2024 at a tertiary referral center (Westmead Hospital, Sydney, Australia) were retrospectively reviewed. All patients experienced at least one episode of VF (defined as a rapid irregular electrical activity > 200 beats per minute with variable waveforms) detected by an implantable cardioverter defibrillator (ICD) or automated external defibrillator and validated by cardiologists within 6 months before ablation were included [8]. Patients with VF preceded by VT, or with a reversible cause, including an electrolyte abnormality, were excluded. VF storm was defined as ≥ 3 separated episodes of VF in 24 h. Patients without SHD, confirmed by diagnostic imaging including magnetic resonance imaging and echocardiography, and the absence of abnormal voltage during electroanatomic mapping (EAM) were excluded. ICM was defined by the presence of coronary artery disease affecting ventricular function. NICM patients were identified as an absence of relevant coronary artery disease and defined according to a previous report [9]. Demographic, procedural, and follow‐up data were retrospectively collected from electronic medical records. The Western Sydney Local Health District Human Research Ethics Committee approved the study.

### 2.1 Mapping and CA Procedure

All procedures were performed under general anesthesia or deep sedation. Anti‐arrhythmic drugs were stopped five half‐lives before ablation, except for amiodarone and in urgent cases. Intravenous heparin (100 units/kg) was given as a bolus after achieving vascular access, with additional boluses to maintain an activated clotting time > 350 s. A quadripolar catheter was deployed in the RV apex, and an intracardiac echocardiography (ICE) catheter was inserted through the femoral vein. An SL3 sheath (Abbott Medical, Chicago, Illinois, USA) was used to insert a decapolar catheter into the coronary sinus. The endocardial LV was mainly accessed transeptally (Large curve Agilis, Abbott), and a retrograde approach with an 8.5 Fr SL0 (Abbott) was used when needed (e.g., mapping at the aortic cusp). The endocardial surface was mapped using the multi‐electrode catheter (DecaNav or Pentarray, Biosense Webster, Diamond Bar, California, USA; or HD grid or Livewire, Abbott) with a three‐dimensional EAM system (CARTO, Biosense Webster, or Ensite Precision or EnsiteX, Abbott) to guide catheter manipulation. In the EAM system and the CardioLab EP Recording System (GE Healthcare, Chicago, Illinois, USA), bandpass filtering was performed at 30–500 Hz for bipolar signals and 2−240 Hz for unipolar signals. An endocardial three‐dimensional shell of chamber geometry was constructed for each ventricle with electrogram recordings. The CARTOSOUND module (SoundStar; Biosense Webster) was used in patients using CARTO system, and a three‐dimensional reconstruction of the RV and LV was created from sequential two‐dimensional ICE slices acquired from the right atrium and ventricle.

CA was performed, including targeting the triggering and/or clinically frequent PVCs, targeting any inducible VTs, and scar homogenization. If triggered or frequent PVCs were present during the procedure, the PVCs were targeted for ablation, utilizing a combination of activation and pace mapping. To induce VT/VF, programmed electrical stimulation was performed from the RV apex, using a 400 ms drive train with up to four extra stimuli beginning at 300 ms and decrementing by 10 ms down to ventricular refractoriness. When induced VTs were hemodynamically tolerated, activation mapping was performed to identify critical isthmus or breakthrough sites. When VTs were not hemodynamically tolerated, ablation targets were identified using pace mapping and mapping of late and/or fractionated potentials. Further scar homogenization was performed by the operator's discretion, based on multiple factors such as the size and distribution of scar, and/or the presence of inducible VT related to scar; Scar homogenization included ablation of bipolar low voltage area (LVZ) and abnormal potentials, as well as unipolar LVZ in the NICM cases. Ablation was performed using an irrigated radiofrequency catheter (ThermoCool STSF, Biosense Webster; TactiCath SE or Tactiflex SE, Abbott) at standard power settings of 30−50 W, aiming for an impedance fall of 10−20 ohms at each site. After the ablation, the inducibility of VT and VF was assessed using programmed extra stimulation from the RV apex with up to four extra stimuli down to ventricular refractoriness, with or without isoprenaline.

### 2.2 Ablation Procedure and Arrhythmogenic Substrate Analysis

The EAM maps during sinus or paced rhythm were assessed to elucidate scar characteristics; Partial substrate maps, which did not cover the entire chamber surface, were excluded from the substrate assessment. The chamber mapped was based on the induced or spontaneously occurring VA characteristics, scar on ICE, and findings on pre‐procedural imaging.

The voltage maps were evaluated based on the criteria previously reported: < 0.5 mV for dense scar and < 1.5 mV for LVZ in bipolar endocardial mapping, and < 5.5 mV and < 8.3 mV for LVZ in RV and LV unipolar endocardial mapping, respectively [10, 11, 12]. The distal Purkinje network was identified by sharp potentials preceding the larger ventricular electrograms during sinus rhythm or during atrial pacing by < 10 ms. An abnormal Purkinje potential was defined as a split or fractionated low‐amplitude signal prior to the local ventricular signal (Supporting Information S1: eFigure 1) [6, 7]. For the LV substrate maps performed during the sinus rhythm or atrial pacing, the bundle potentials, the distal Purkinje networks, and abnormal Purkinje potentials were manually marked, retrospectively, after the procedures by an electrophysiologist. The number of abnormal Purkinje potentials was quantified as a proportion of the mapped surface points. The areas of LVZ and dense scar area were calculated and quantified as a proportion to the total surface area. Additionally, the ratio between bipolar and unipolar LVZ and the proportion of bipolar dense scar to the bipolar LVZ were calculated.

### 2.3 Outcomes

The follow‐up duration was defined as the period from the index VF ablation procedure to the last clinical follow‐up or death. Clinical follow‐up included a review of hospital and outpatient clinic records and ICD remote‐monitoring data. The primary outcome was VF recurrence after single and multiple ablations detected by ICD. The secondary outcome included VF burden, in‐hospital death, and VA recurrence. VF burden was described as the number of VF episodes in the preceding 6 months compared to the 6 months after the ablations. The VA recurrence was defined as VT lasting more than 30 s or sustained VT or VF requiring ICD therapies. VF zones on ICDs were programmed based on the expert consensus statement, but individually modified by the physician's discretion to minimize ICD shocks under consciousness [13].

### 2.4 Comparison With Matched Cohort in NICM Patients

For the substrate comparison, each NICM case was matched 1:1 to a control NICM cohort who underwent VT ablation without a history of VF. The aim of this comparison was to investigate the VF‐specific substrate characteristics in comparison with the arrhythmogenic substrate in NICM patients with monomorphic VT. Among the NICM patients who underwent VT ablation during the same time, 1:1 matching was performed according to age (within 5 years), gender, and the closest left ventricular ejection fraction.

### 2.5 Statistical Analysis

The Statistical Package for the Social Sciences version 29 (IBM Corp., Armonk, NY) was used for analysis. Continuous variables were expressed as the mean ± standard deviation or median and 25%–75% interquartile range (IQR), and compared using *t*‐test or Mann–Whitney *U* test, as appropriate. Categorical variables were expressed as frequencies and percentages and compared using Fisher's exact test. VF burden before and after ablation was compared with the Wilcoxon signed‐rank test. Event‐free survival was assessed using Kaplan–Meier analysis with log‐rank test, and Cox regression was used to estimate hazard ratios (HR) and 95% confidence intervals (CI). Since the NICM group included various types of cardiomyopathies, a sensitivity analysis was performed after excluding non‐idiopathic NICM patients (cardiac sarcoidosis, hypertrophic cardiomyopathy, and valvular cardiomyopathy). Thus, ICM and idiopathic NICM were compared in the sensitivity analysis. A two‐tailed *p* < 0.05 was considered significant.

## 3 Results

### 3.1 Baseline Characteristics

Forty‐five patients met the inclusion criteria and were included in the present analysis: 17 ICM and 28 NICM patients. Of 28 NICM patients, underlying heart disease was classified as idiopathic NICM in 20 patients, valvular heart disease in four patients, cardiac sarcoidosis in three patients, and hypertrophic cardiomyopathy in one patient. Overall, the mean age was 60 ± 15 years (64 ± 14 years in ICM vs. 58 ± 15 years in NICM, *p* = 0.14), and 87% were male (82% in ICM vs. 89% in NICM, *p* = 0.66). The ICM group had a lower left ventricular ejection fraction compared to the NICM group (32 ± 12% vs. 45 ± 14%, *p* < 0.01). The proportions of concomitant VT were comparable between the two groups (53% vs. 50%, *p* = 1.00) (Table 1).

**Table 1:** Baseline characteristics.

	Overall (*n* = 45)	ICM (*n* = 17)	NICM (*n* = 28)	*p* value
Age, years old	60.0 ± 14.7	64.1 ± 13.6	57.5 ± 15.0	0.14
Male	39 (86.7)	14 (82.4)	25 (89.3)	0.66
LVEF, %	40.5 ± 14.4	31.9 ± 11.5	45.5 ± 13.7	< 0.01
BMI, kg/m^2^	29.9 ± 7.8	27.0 ± 5.3	31.6 ± 8.5	0.06
Post CABG	6 (13.3)	6 (35.3)	0 (0)	< 0.01
ICD	39 (86.7)	13 (76.5)	26 (92.9)	0.13
VF storm	12 (26.7)	6 (35.3)	6 (21.4)	0.33
VT	23 (51.1)	9 (52.9)	14 (50)	1.00
Beta‐blocker	34 (75.6)	13 (81.3)	21 (75)	0.72
AAD	27 (60.0)	12 (75)	15(53.6)	0.21
Amiodarone	17 (37.8)	8 (47.1)	9 (32.1)	0.36
Sotalol	7 (15.6)	2 (11.8)	5 (17.9)	0.69

*Note:* Data are reported as mean ± standard deviation or *n* (%).

Abbreviations: AAD, anti‐arrhythmic drug; BMI, body mass index; CABG, coronary artery bypass grafting; ICD, implantable cardioverter defibrillator; LVEF, left ventricular ejection fraction; VF, ventricular fibrillation; VT, ventricular tachycardia.

### 3.2 Substrate Characteristics

Fifteen (88%) LV substrate maps covering the whole chamber were created in the ICM group, and 26 (93%) were created in the NICM group. In the NICM group, the bipolar and unipolar LVZ were observed in 16 patients (67%) and 21 patients (88%), respectively, and both the bipolar and unipolar LVZ were present in all 15 maps in the ICM group (Table 2). When compared to the NICM group, the ICM group had a larger LVZ percentage of total surface area in both bipolar voltage (median 30% [IQR 26, 41] vs. 12% [5, 24], *p* < 0.01) and unipolar voltage (median 61% [33, 88] vs. 27% [14, 48], *p* < 0.01). The proportion of the dense scar in the bipolar LVZ and the bipolar‐unipolar ratio were similar (Figure 1). LV substrate mapping was performed during sinus rhythm or atrial pacing in 10 ICM and 15 NICM patients, for which the distributions of the bundle potentials, the distal Purkinje networks, and the abnormal Purkinje potentials were assessed. The prevalence of the abnormal Purkinje potentials was similar between the ICM and NICM groups (median 2.9% [IQR 2.4, 4.3] vs. 2.4% [0.9, 3.2], *p* = 0.28; Figure 1). Sensitivity analysis was performed by excluding non‐idiopathic NICM patients, and substrate characteristics were compared between ICM and idiopathic NICM patients. The findings were consistent with the main analysis (Supporting Information S1: eFigure 2). RV substrate mapping was performed in 10 patients (36%) in the NICM group, while no RV mapping was performed in the ICM group. Of the 10 RV substrate maps in the NICM group, three had the bipolar LVZ, and five had the unipolar LVZ.

**Table 2:** Procedural data.

	ICM (*N* = 17)	NICM (*N* = 28)	*p* value
LV voltage map	15 (88.2)	26 (92.9)	0.63
Total mapping points	3609 ± 3064	2343 ± 1209	0.15
Bipolar LVZ	15 (100)	16 (66.7)	0.02
Unipolar LVZ	15 (100)	21 (87.5)	0.27
RV voltage map	0 (0)	10 (35.7)	< 0.01
Total mapping points	—	1521 ± 982	—
Bipolar LVZ	—	3 (30.0)	—
Unipolar LVZ	—	5 (50.0)	—
LV ablation	17 (100)	27 (96.4)	1.00
Ablation at papillary muscles	12 (70.6)	10 (35.7)	0.03
RV ablation	2 (11.8)	7 (25.0)	0.45
Septum ablation	15 (88.2)	25 (89.3)	1.00
Scar homogenization	13 (76.5)	16 (57.1)	0.22
PVC ablation	7 (41.2)	11 (39.3)	1.00
Purkinje area ablation	13 (76.5)	8 (28.6)	< 0.01
Ablation area, cm^2^	37.7 ± 23.9	16.8 ± 12.6	< 0.01
Pre‐induction	15 (88.2)	27 (96.4)	0.55
Pre‐VT induced	7 (41.2)	20 (71.4)	0.11
Number of VTs	2.1 ± 1.8	2.5 ± 1.8	0.70
Pre‐VF induced	5 (29.4)	8 (28.6)	1.00
Post‐induction	11 (64.7)	24 (85.7)	0.14
Post‐VF induced	4 (23.5)	8 (28.6)	1.00
ECMO use	4 (23.5)	0 (0)	0.02
VT non‐inducibility at the end	10 (58.8)	17 (60.7)	1.00
Anticipated AVB	2 (11.8)	6 (21.4)	0.69
Procedure‐related complication	4 (23.5)	0 (0)	0.02

*Note:* Data are reported as mean ± standard deviation or *n* (%).

Abbreviations: AVB, atrioventricular block; ECMO, extracorporeal membrane oxygenation; LV, left ventricle; LVZ, low voltage zone; RV, right ventricle; VF, ventricular fibrillation; VT, ventricular tachycardia.

**Figure 1:**
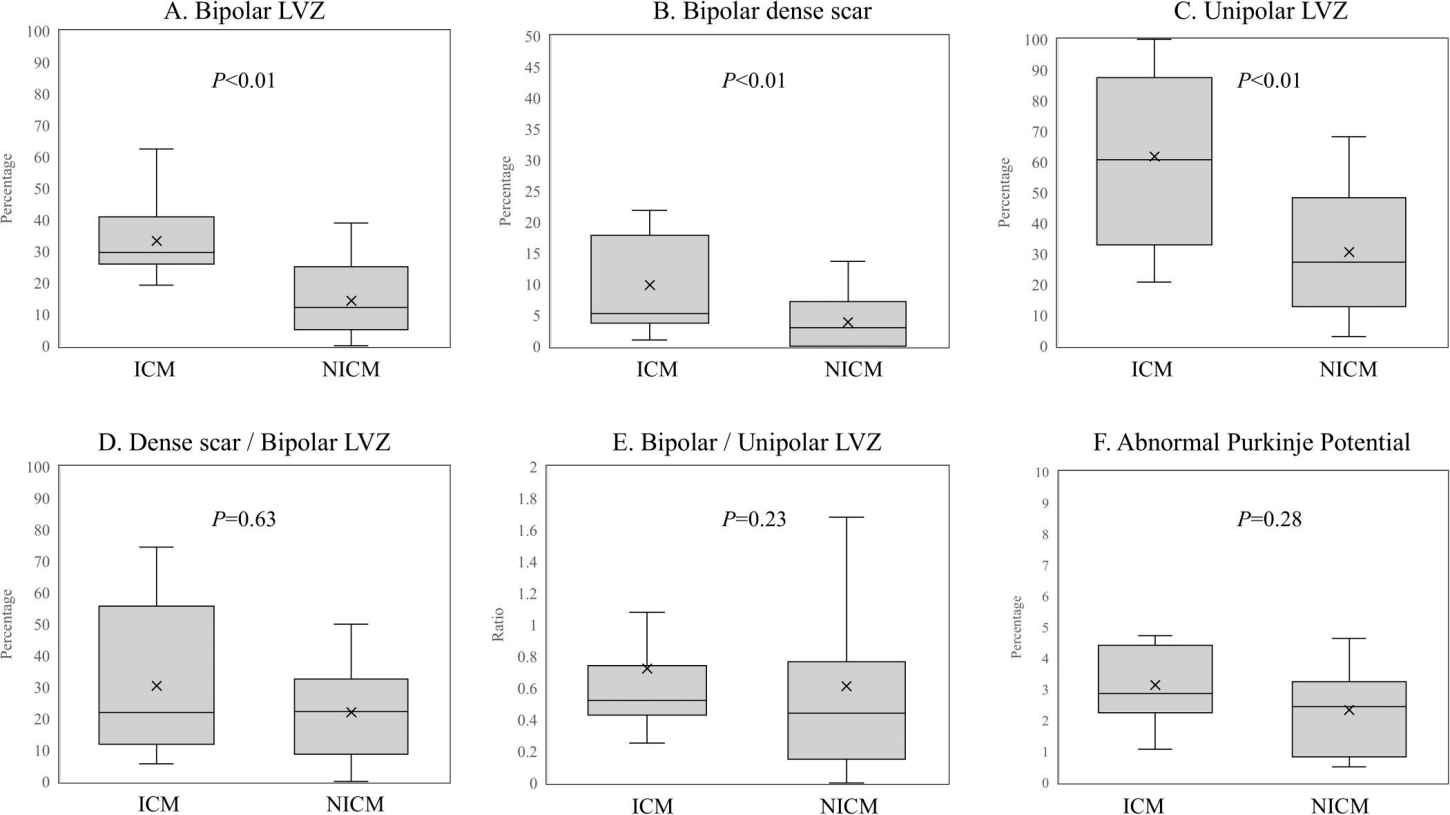
Comparison of the left ventricular endocardial substrate characteristics between ischemic cardiomyopathy and non‐ischemic cardiomyopathy. (A−C) Ischemic cardiomyopathy (ICM) was associated with a larger bipolar low voltage zone (LVZ), bipolar dense scar, and unipolar LVZ. (D−F) The proportion of the bipolar dense scar to the bipolar LVZ, the ratio between unipolar LVZ and bipolar LVZ, and the prevalence of abnormal Purkinje potentials were comparable. ICM, ischemic cardiomyopathy; LVZ, low voltage zone; NICM, non‐ischemic cardiomyopathy.

### 3.3 Ablation Strategy

PVC ablation was performed in seven ICM patients (41%) and 11 NICM patients (39%) (*p* = 1.00), among whom additional scar homogenization was performed in four patients in the ICM group and five patients in the NICM group. Ablation strategies in the ICM and NICM groups are shown in Supporting Information S1: eFigures 3–4.

In seven ICM cases (41%), PVCs were targeted for the ablation; three were VF‐triggering PVCs, whereas four were clinically frequent PVCs. The origins of targeted PVCs in the ICM group were from papillary muscles in two, adjacent to the fascicle in four, and aorto‐mitral continuity in one. Of seven targeted PVCs, five (71%) arose from the sites where the distal Purkinje network or abnormal Purkinje potentials were confirmed in the substrate maps: one was from the distal Purkinje network at the terminal portion of the posterior fascicle, and the other four were from the abnormal Purkinje potentials, which were located at bipolar scar border zone in three and at bipolar dense scar zone in one.

In 11 NICM cases (39%), 17 PVCs were targeted for the ablation; among which seven were VF‐triggering PVCs, whereas 10 were clinically frequent PVC. The origins of targeted PVCs in the NICM group were from papillary muscles in seven, adjacent to the posterior fascicle in one, septum in six, and other LV myocardium in three: (1) LV summit at normal bipolar and unipolar voltage, (2) the basal inferolateral LV wall at bipolar and unipolar scar, (3) lateral mitral annulus at normal bipolar and unipolar voltage zone. Of 17 targeted PVCs, six arose from the site where the distal Purkinje network or abnormal Purkinje potentials were present: four were from the distal Purkinje network at the papillary muscles or the terminal portion of the posterior fascicle, and the other two were from the abnormal Purkinje potentials located at the edge of the bipolar scar in the mid‐septum and anterolateral papillary muscles. The representative NICM case with PVC that arose from the abnormal Purkinje area is shown in Figure 2. The details of the targeted PVCs is shown in Supporting Information S1: eTable 1.

**Figure 2:**
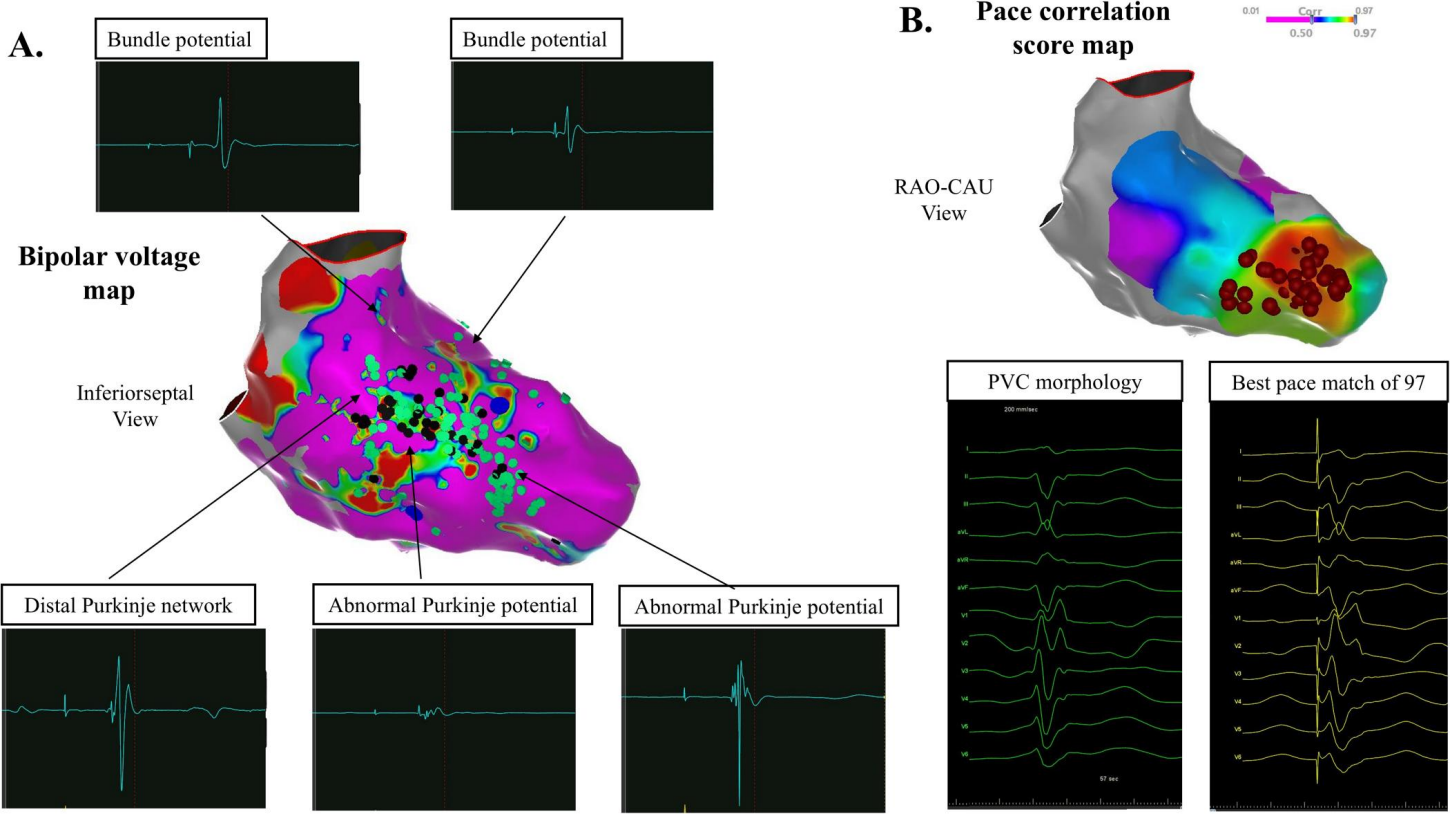
Representative case with premature ventricular complex ablation in a hypertrophic cardiomyopathy patient. (A) There is a patchy low‐voltage zone in the inferior septum. The black and green dots represent abnormal Purkinje potentials and the distal Purkinje network, respectively. (B) The clinical premature ventricular complex (PVC) appeared during the procedure. The best pace map score of 97% at the inferior septum, where the abnormal Purkinje potentials were present in the substrate map during sinus rhythm. Ablations at this site eliminated the PVC, and the patient has remained free from ventricular fibrillation throughout the past year. PVC, premature ventricular complex.

Among 10 ICM patients and 17 NICM patients in whom the PVCs were not targeted, nine ICM and 11 NICM patients underwent scar homogenization, whereas the rest of the patients (*n* = 7) only underwent ablation targeting inducible VT (Supporting Information S1: eFigures 2–3). Overall, 13 ICM patients (81%) and 16 NICM patients (57%) underwent scar homogenization. The total ablation area was significantly larger in the ICM group, compared to the NICM group (38 ± 24 vs. 17 ± 13 cm^2^, *p* < 0.01). Scar homogenization included the Purkinje potential area, where the distal Purkinje network or the abnormal Purkinje potentials were present in 10 ICM patients (77%) and seven NICM patients (44%). The representative cases with scar homogenization in the NICM and ICM groups are shown in Figures 3 and 4, respectively.

**Figure 3:**
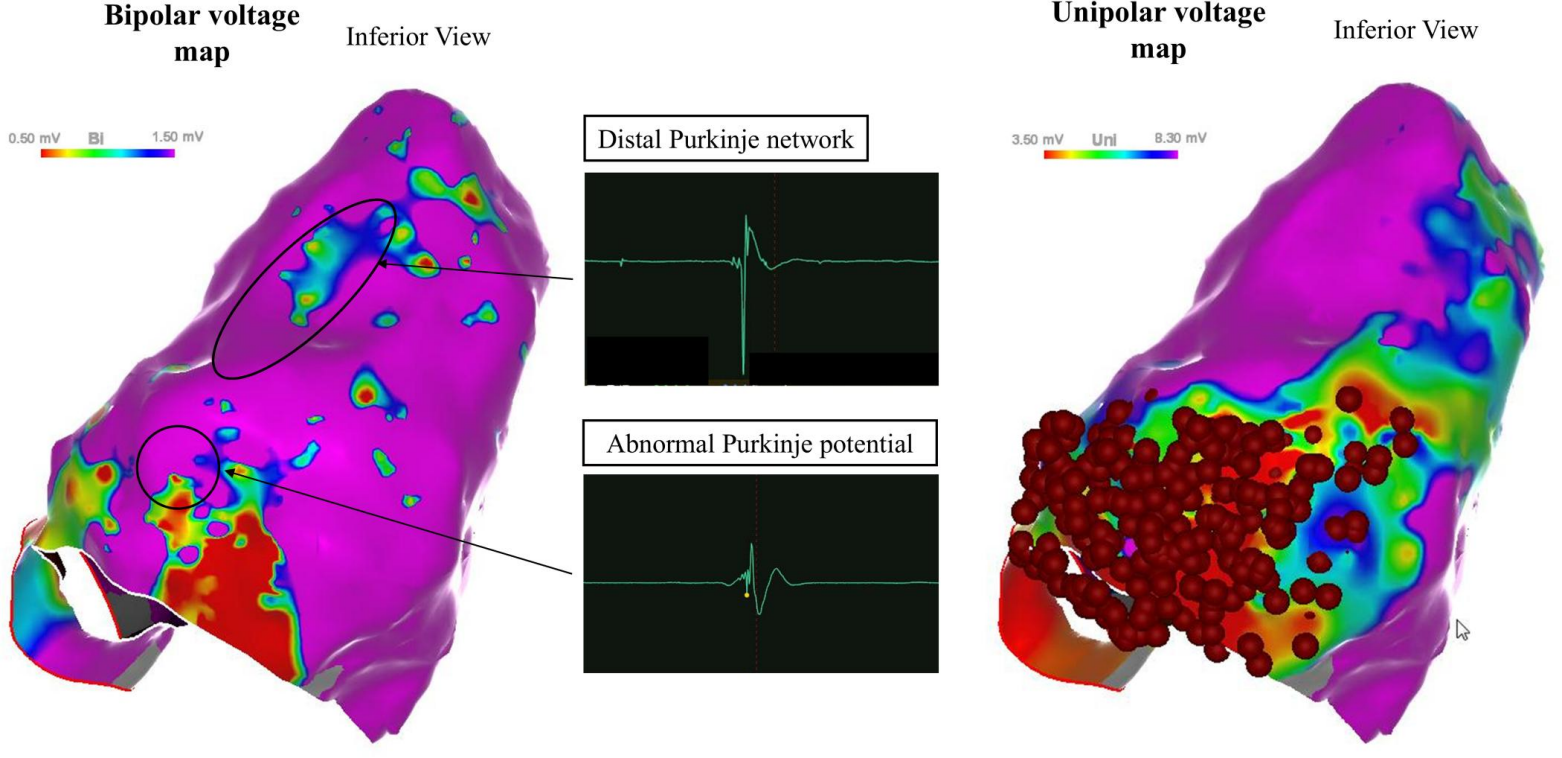
Representative case with scar homogenization in a non‐ischemic cardiomyopathy patient. A bipolar low voltage zone (LVZ) is present at the basal inferior wall, and abnormal Purkinje potentials are present at the scar border zone close to the distal Purkinje network. Unipolar LVZ is more extensive, which implies an intramural or epicardial substrate. Scar homogenization was performed targeting the unipolar LVZ. Red dots indicate ablation lesions.

**Figure 4:**
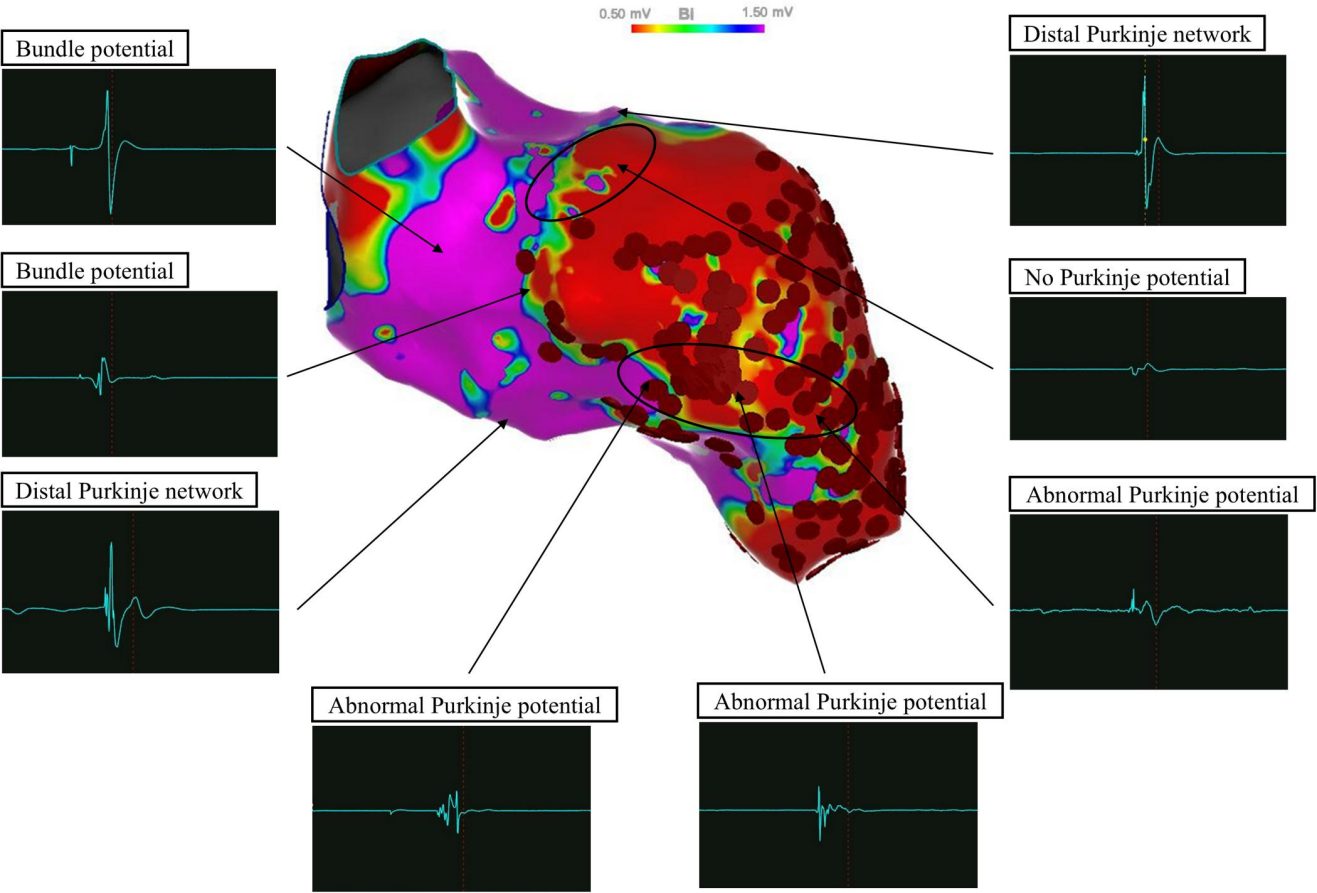
Representative case with scar homogenization in an ischemic cardiomyopathy patient. The bipolar voltage map represents a large anterior scar extending to the apex. The abnormal Purkinje potentials are broadly present at the anterior septum. Scar homogenization mainly targets the abnormal Purkinje. Red dots indicate ablation lesions. Notably, no abnormal Purkinje potential was observed along the left anterior fascicle region in this case.

Ablation at the septum was delivered in 15 ICM patients (88%) and 25 NICM patients (89%; *p* = 1.00). Ablation at the basal septum substrate near the conduction system was required and resulted in anticipated atrioventricular block in two ICM patients and six NICM patients (*p* = 0.69), and left bundle branch block was seen in one NICM patient. Ablation at the papillary muscles was delivered more frequently in the ICM group, compared to the NICM group (71% vs. 36%, *p* = 0.03; Table 2). Epicardial ablation was performed in two NICM patients in the index procedure due to an epicardial substrate identified by preprocedural imaging and previously failed endocardial ablation, respectively. Five NICM patients underwent epicardial ablation during repeated procedures to target the epicardial substrate after the failed endocardial ablation in the index procedure.

### 3.4 Procedural Data

At the beginning of the cases, VF was induced in five ICM patients (29%) and eight NICM patients (29%), and VTs were induced in six ICM patients (35%) and 20 NICM patients (71%) by programmed electrical stimulation (*p* = 1.00 and *p* = 0.11, respectively). After the ablation, VF was inducible in four ICM patients (24%) and eight NICM patients (29%) (*p* = 1.00; Table 2). Extracorporeal membrane oxygenation was utilized in four ICM patients (24%) but none of the NICM patients (*p* = 0.02). Four procedural‐related complications (24%) occurred in the ICM patients, whereas none in the NICM patients (*p* = 0.02): (1) in‐hospital death due to VA recurrence, (2) limb ischemia related to the extracorporeal membrane and subsequent in‐hospital death due to cardiogenic shock, (3) thrombosis related to the extracorporeal membrane, and (4) in‐hospital death due to cardiogenic shock after extracorporeal membrane removal.

### 3.5 Follow‐Up Data

Three ICM patients (18%) died during the index hospitalization, whereas none in the NICM (*p* = 0.048). All three patients presented with VF storm; two of them required rescue extracorporeal membrane support before the procedures and died due to cardiogenic shock after withdrawal of the extracorporeal membrane support, and the other patient experienced recurrence soon after the procedure, and palliative care was taken afterward. None of the in‐hospital mortality was related to the ablation‐related complication.

Of the 42 discharged patients, 12 (29%) continued anti‐arrhythmic drugs post‐procedure, with similar proportions in ICM (29%) and NICM (29%) (*p* = 1.00). One ICM patient died 273 days after the index procedure due to a non‐cardiovascular cause. The remaining patients (*n* = 41) had at least 1 year of follow‐up. One year after the last procedure, anti‐arrhythmic drugs were taken in 54% of the ICM group and 39% of the NICM group (*p* = 0.50). Two ICM patients (12%) and four NICM patients (14%) experienced VF recurrence within 1‐year follow‐up after the last procedure (*p* = 1.00).

The median (IQR) follow‐up was 713 (420–1371) days post‐ablation procedure. After the first ablation, 17 patients (7 ICM, 10 NICM) had VF recurrence at a median of 156 days (IQR 13–396), and eight patients (3 ICM, 5 NICM) underwent repeat ablation. Overall, 11 patients (4 ICM, 7 NICM) experienced VF recurrence after multiple procedures. VF‐free survival after single and multiple ablations was comparable between groups (log‐rank *p* = 0.69 and 0.93, respectively; Figure 5). Also, the VA‐free survival rate after the first VF ablation was similar between the two groups (Supporting Information S1: eFigure 5). Sensitivity analysis comparing ICM and idiopathic NICM patients showed consistent results, showing comparable VF‐survival rate after single and multiple ablations in the two groups (Supporting Information S1: eFigure 6). When comparing patients with and without concomitant VT, the VF‐free survival after single and multiple were comparable (log‐rank *p* = 0.12 and 0.93, respectively; Figure 5). The VF‐free survival rate after a single ablation was not different regardless of whether PVC could be targeted during the procedures (log‐rank *p* = 0.79, HR 1.14, 95% CI 0.44−2.96; Supporting Information S1: eFigure 7).

**Figure 5:**
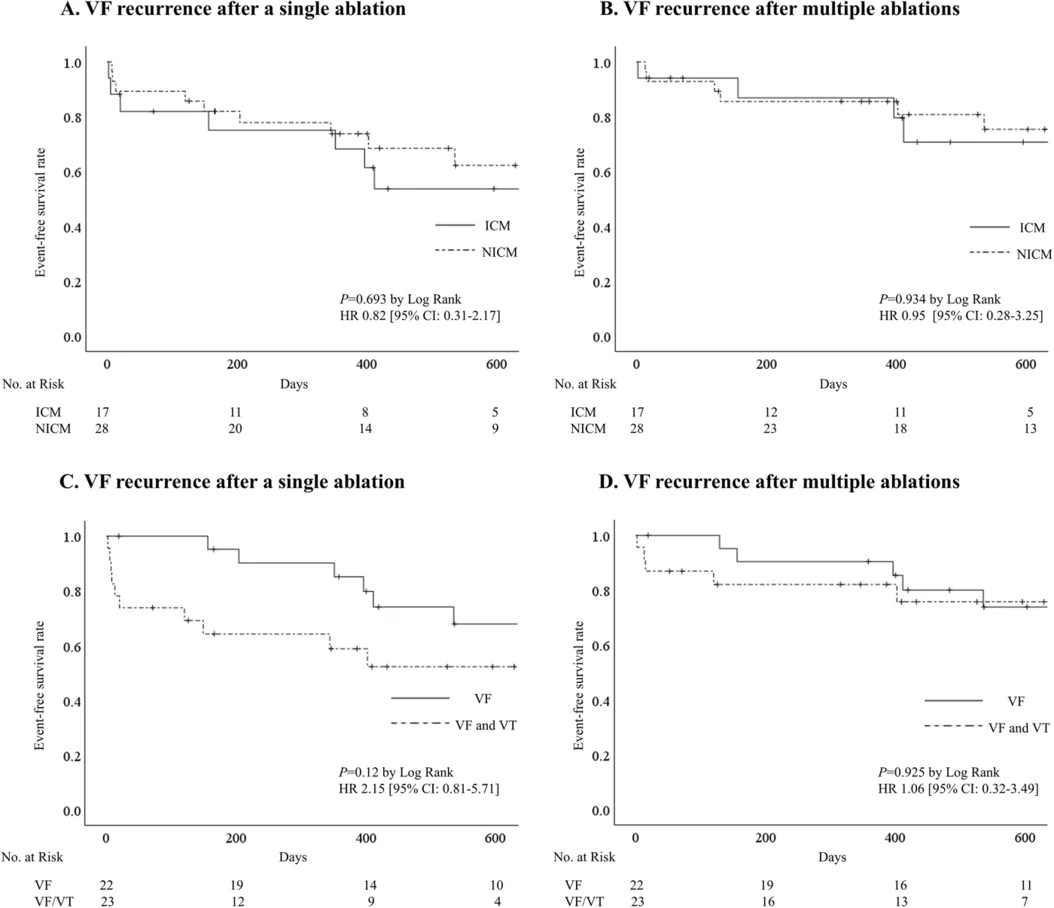
Event‐free survival analysis for the ventricular fibrillation recurrence. Cumulative incidence of ventricular fibrillation recurrence after a single or multiple ablations was similar between ischemic and nonischemic cardiomyopathy (panel A and B), and also between patients with or without concomitant ventricular tachycardia (panel C and D). CI, confidence intervals; HR, hazard ratio; ICM, ischemic cardiomyopathy; NICM, nonischemic cardiomyopathy; VF, ventricular fibrillation.

### 3.6 Six‐Month Burden Before and After Ablation

The 6‐month VF burden after a single ablation was available in 38/45 patients (87%): reasons for absent burden data included in‐hospital death in three patients and repeated ablation within 30 days in four patients. Any reduction, greater than 50% reduction, and complete reduction were achieved after a single ablation in 36 patients (95%), 36 patients (95%), and 33 patients (87%), respectively. The median (IQR) 6‐month VF burden before and after the ablation was 2 (1−5) and 0 (0−0) (*p* < 0.01), respectively.

Four patients required repeated ablation within 30 days after the index ablation due to VF recurrence: a patient with dilated cardiomyopathy had VF recurrence 5 days after PVC ablation and scar homogenization, and underwent repeated ablation; however, the patient experienced VF and VT storm 49 days after the second ablation and underwent a third ablation again including epicardial ablation. Thereafter, no VF recurrence has not occurred for 4 years. The three other patients (1 idiopathic NICM and 2 ICM) had no VF episode after a redo procedure. After multiple ablations, any reduction, greater than 50% reduction, and complete reduction were achieved in 95%, 95%, and 88%, respectively.

Two patients who did not achieve any VF burden reduction are described as follows: (1) a patient with dilated cardiomyopathy who presented with multiple VF episodes and underwent VF ablation. The LV substrate map revealed LVZ at the basal septum, with a larger LVZ area in the unipolar voltage map compared to the bipolar voltage map. Since no VF‐triggering or frequent PVC could be identified during the case, scar homogenization was performed; however, the patient experienced four VF episodes in the 30 days post‐ablation. The patient was subsequently treated with amiodarone afterward without further ablation. (2) A patient with valvular cardiomyopathy had a single VF episode before ablation. VF‐triggering PVC ablation was performed at the posteromedial papillary muscle, along with scar homogenization that included the abnormal Purkinje potential area. VF non‐inducibility was achieved after the procedure; however, VF episodes recurred post‐ablation. Six months later, the patient underwent a repeated ablation, targeting the VF‐triggering PVC from the anterolateral papillary muscle. Although two isolated VF episodes occurred within a year following the second ablation, no VF or VT episode has been reported in the 4 years since.

### 3.7 Comparison to the Matched NICM Cohort

Twenty‐eight matched NICM patients who underwent VT ablation without a history of VF had the same gender ratio and similar age and left ventricular ejection fraction, and were compared to the NICM group with VF. The baseline characteristics are shown in Supporting Information S1: eTable 2. When comparing the LV substrate map, the bipolar and unipolar LVZ areas, their ratio, the proportion of dense scar, and the prevalence of abnormal Purkinje potentials were all comparable (Supporting Information S1: eFigure 8).

## 4 Discussion

The current study investigated SHD patients with VF undergoing CA. Our data suggests that ablation targeting PVCs and/or scar homogenization can successfully ameliorate VF episodes, regardless of the underlying cardiomyopathy (ICM or NICM). The important findings from our study were:

1. VF‐burden reduction and freedom from VF recurrence could be achieved in patients with ICM and NICM by targeting the triggering or clinically frequent PVC (where possible), empiric scar homogenization, or a combination of PVC ablation and scar homogenization.
2. There was no discernible difference in frequency of abnormal Purkinje potentials when comparing ICM and NICM patients with VF, nor were there any differences in NICM who presented with VF, compared to those with VT in a matched cohort comparison.
3. Three ICM patients (18%) experienced in‐hospital death due to disease severity and progression, while none in the NICM group, which represents the different severity and acute prognosis in patients with VF between ICM and NICM.

In patients with ICM, ablation targeting a VF‐triggering PVC is regarded as a reasonable therapy; the triggering PVC often arises from preceding Purkinje potentials, especially in ICM patients [4, 14]. Komatsu et al. reported that more than 90% of the triggering PVCs originated from the LV septum or papillary muscle when they studied 110 VF storm patients after myocardial infarction. We reported our experience, including 45 consecutive cardiomyopathy patients with VF and, notably, 28 NICM patients. In 18 patients in whom PVCs were ablated, the majority of which originated from LV papillary muscle, adjacent to the fascicle or septum in both NICM and ICM (Supporting Information S1: eTable 1). The origin in the Purkinje area, which arose from the regions with the distal Purkinje networks or the abnormal Purkinje potentials, was more common in the ICM patients than NICM (71% vs. 35%). Our findings in the ICM group were generally consistent with the previous study [4]. Furthermore, our study showed that the origin of PVCs in NICM was mainly from the papillary muscles or septum, often related to the Purkinje area.

However, targeting the PVC is not always feasible during VF ablation due to the infrequency or absence of the triggering PVCs. The alternative strategy of targeting the arrhythmogenic substrate has been explored. Hasegawa et al. reported a long‐duration VF related to ICM on extracorporeal membrane oxygenation support terminated by radiofrequency applications to Purkinje activation, which suggested that the Purkinje network plays a pivotal role in maintaining VF [5]. A randomized controlled trial that investigated ICM patients with unstable VT and/or VF revealed that substrate‐based ablation reduced the incidence of ICD therapy [1]. More recently, Kitamura et al. investigated the efficacy of local abnormal ventricular activity elimination for SHD patients who had both VT and VF, and demonstrated its association with reduced VF burden [8]. Nakamura et al. reported the feasibility of substrate modification targeting low voltage scar in addition to triggering PVC ablation in 19 patients with SHD, including 12 NICM [15]. Targeting ventricular scar and abnormal potentials appears feasible to prevent both VT and VF in SHD, as triggering PVCs and VF drivers may colocalize with the VT substrate in scar and border zones. However, the ablation strategy, including substrate modification for NICM patients with VF, has not been fully investigated. We included 28 NICM patients with VF, and 57% underwent scar homogenization. The reduction of VF burden was achieved in most cases in both ICM and NICM after ablation. Interestingly, the long‐term outcomes between the ICM and NICM groups were comparable, implying that targeting the trigger and substrate might be a reasonable strategy even in NICM patients.

We also assessed the characteristics of the arrhythmogenic substrate. Krummen et al. mapped VF with basket catheters in six SHD patients and identified 20 substrates related to VF, 85% linked to bipolar or unipolar abnormalities [16]. Haissaguerre et al. used body‐surface mapping in SHD patients and showed that VF was mainly sustained by the Purkinje network and abnormal electrograms (bipolar LVZ and fractionated potentials). They reported that ablation of abnormal electrograms plus PVCs reduced VF burden [6]. Bode et al. reported fascicular modification targeting the distal Purkinje network and abnormal Purkinje potentials prevented VF recurrence in five of seven NICM patients over 2 years [7]. In our study, ICM patients had larger scar and ablation areas, but the proportion of abnormal Purkinje potentials was similar to NICM. Matched control NICM patients without a history of VF, who had previously undergone VT ablation, were used to compare the substrate characteristics with those of NICM patients with VF, to investigate VF‐specific substrate among NICM patients. However, compared with matched NICM without VF, NICM with VF showed no distinct substrate differences. This finding implies that an empiric scar homogenization approach may be warranted to prevent VF. Further studies to elucidate the arrhythmogenic substrate that specifically causes VF are essential.

### 4.1 Limitations

This retrospective study has several limitations, including potential selection bias, small sample size, and absence of a medical therapy control group. The decision‐making process for the substrate ablation strategy was not standardized due to the retrospective nature of this study, which is a major limitation of our analysis. Scar homogenization overlapped Purkinje substrate in 77% of ICM and 44% of NICM cases, making it difficult to separate the effects of Purkinje substrate modification from scar homogenization. In addition, a lower rate of Purkinje substrate ablation in the NICM group might have affected the outcome, since Purkinje substrate ablation could have decreased the risk of VF recurrence. Larger prospective studies with a systematic ablation strategy are needed to confirm the efficient CA strategy for VF.

## 5 Conclusion

For patients with VF and the NICM, targeting arrhythmogenic substrate along with triggering or clinically frequent PVC may be a reasonable approach, with similar outcomes to patients with ICM and recurrent VF. Further studies are warranted to investigate the specific VF mechanism and the optimal targets for ablation (Central illustration 1).

**Central illustration 1:**
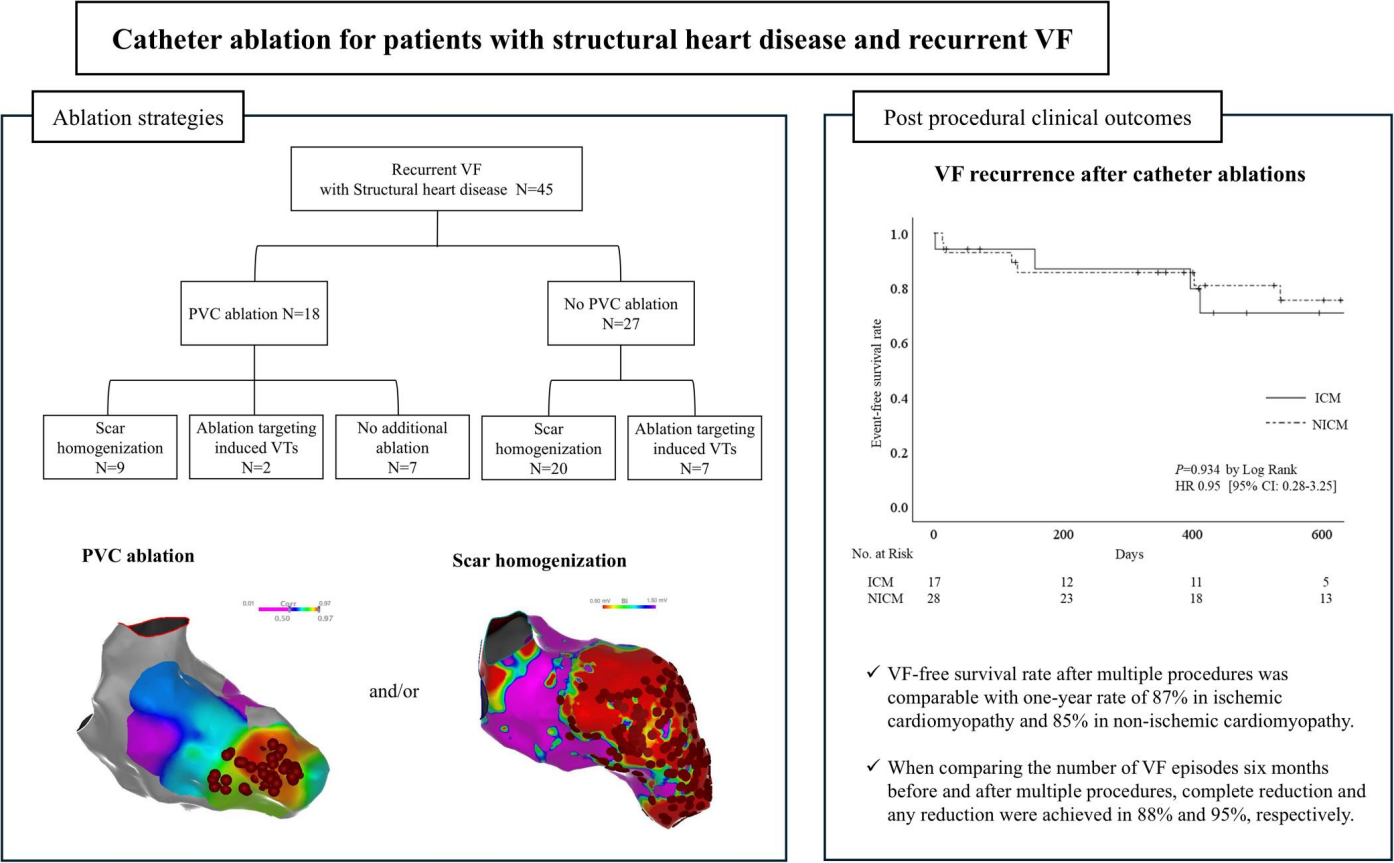
CI, confidence intervals; HR, hazard ratio; ICM, ischemic cardiomyopathy; NICM, non‐ischemic cardiomyopathy; PVC, premature ventricular complex; VF, ventricular fibrillation.

## Funding

The authors have nothing to report.

## Disclosure

The authors have nothing to report.

## Supporting information


**Figure S1:** Distal Purkinje network and abnormal Purkinje potential in an electro‐anatomical map. **Figure S2:** Sensitivity analysis: comparison of the left ventricular endocardial substrate characteristics between ischemic cardiomyopathy and idiopathic non‐ischemic cardiomyopathy. **Figure S3:** Flow diagram of ablation strategy for ischemic cardiomyopathy. **Figure S4:** Flow diagram of ablation strategy for non‐ischemic cardiomyopathy. **Figure S5:** Event‐free survival analysis for the ventricular arrhythmia recurrence in ischemic cardiomyopathy and non‐ischemic cardiomyopathy. **Figure S6:** Sensitivity analysis: event‐free survival analysis for the ventricular fibrillation recurrence in ischemic cardiomyopathy and idiopathic non‐ischemic cardiomyopathy. **Figure S7:** Event‐free survival analysis for the ventricular fibrillation recurrence in patients with and without premature ventricular complex ablation. **Figure S8:** Comparison of the substrate characteristics between non‐ischemic cardiomyopathy with and without ventricular fibrillation. **Table S1:** Details of targeted premature ventricular complexes. **Table S2:** Comparison between non‐ischemic cardiomyopathy patients with and without ventricular fibrillation.

## Data Availability

The data that support the findings of this study are available from the corresponding author upon reasonable request.
