# Supplementary material for: Clinical Outcomes After Catheter Ablation in Patients With Structural Heart Disease and Ventricular Fibrillation
Source: J Cardiovasc Electrophysiol. 2026 Aug 30;37(11):2651–61. doi: 10.1111/jce.70488 (PMC13768790; doi:10.1111/jce.70488)
Supplement: Supplementary file 1 [file JCE-37-2651-s001.docx]

**Supplemental materials**

**eTable 1.** Details of targeted premature ventricular complexes

**eTable 2.** Comparison between non-ischemic cardiomyopathy patients with and without ventricular fibrillation

**eFigure 1.** Distal Purkinje network and abnormal Purkinje potential

**eFigure 2.** Sensitivity analysis: comparison of the left ventricular endocardial substrate characteristics between ischemic cardiomyopathy and idiopathic non-ischemic cardiomyopathy

**eFigure 3.** Flow diagram of ablation strategy for ischemic cardiomyopathy

**eFigure 4.** Flow diagram of ablation strategy for non-ischemic cardiomyopathy

**eFigure 5.** Event-free survival analysis for the ventricular arrhythmia recurrence in ischemic cardiomyopathy and non-ischemic cardiomyopathy

**eFigure 6.** Sensitivity analysis: event-free survival analysis for the ventricular fibrillation recurrence in ischemic cardiomyopathy and idiopathic non-ischemic cardiomyopathy

**eFigure 7.** Event-free survival analysis for the ventricular fibrillation recurrence in patients with and without premature ventricular complex ablation.

**eFigure 8.** Comparison of the substrate characteristics between non-ischemic cardiomyopathy with and without ventricular fibrillation

**eTable 1. Details of targeted premature ventricular complexes**

| Etiology  Patient no. | Type | Origin | V1 pattern | Axis | QRS  Width, ms | Coupling  interval, ms | Bipolar  voltage | Unipolar  voltage | Purkinje  area |
| --- | --- | --- | --- | --- | --- | --- | --- | --- | --- |
| ICM1 | frequent | Anterolateral PM | RB | superior | 156 | 509 | scar | scar | yes |
| ICM2 | trigger | Posterior fascicle | rS | superior | 127 | 377 | scar | scar | yes |
| ICM3 | trigger | Anterior fascicle | RB | inferior | 126 | 345 | normal | scar | yes |
| ICM4 | frequent | Posteromedial PM | RB | superior | 173 | 646 | scar | scar | NA*^2^ |
| ICM5 | frequent | Posterior fascicle | qR | inferior | 115 | 498 | NA*^1^ | NA*^1^ | yes |
| ICM6 | frequent | Aorto-mitral continuity | qR | inferior | 120 | 392 | scar | scar | no |
| ICM7 | trigger | Posterior fascicle | RB | superior | 142 | 595 | dense scar | scar | yes |
| NICM1 | frequent | Posteromedial PM | rS | superior | 170 | 539 | scar | scar | NA*^2^ |
| NICM1 | frequent | Anterolateral PM | rS | inferior | 172 | 543 | normal | normal | NA*^2^ |
| NICM1 | frequent | Para Hisian region | rS | inferior | 172 | 640 | NA*^1^ | NA*^1^ | NA*^2^ |
| NICM2 | trigger | Anterolateral PM | RB | inferior | 150 | 480 | normal | scar | yes |
| NICM3 | trigger | Mid-anteroseptum | RB | inferior | 139 | 390 | normal | normal | yes |
| NICM4 | trigger | Posteromedial PM | RB | superior | 126 | 455 | scar | scar | yes |
| NICM5 | frequent | Lateral mitral annulus | RB | inferior | 179 | 603 | normal | normal | no |
| NICM6 | trigger | Basal inferolateral | RB | superior | 146 | 595 | scar | scar | no |
| NICM7 | frequent | Basal septum | QS | superior | 118 | 609 | dense scar | scar | NA*^2^ |
| NICM7 | frequent | Basal inferoseptum | qR | discordance | 126 | 419 | dense scar | scar | NA*^2^ |
| NICM8 | trigger | Basal inferoseptum | RB | superior | 111 | 490 | scar | scar | No |
| NICM8 | frequent | Posteromedial PM | RB | superior | 118 | 536 | normal | scar | Yes |
| NICM8 | frequent | Anterolateral PM | RB | inferior | 139 | 487 | normal | scar | Yes |
| NICM9 | frequent | Basal septum | RB | inferior | 144 | 574 | NA*^1^ | NA*^1^ | NA*^2^ |
| NICM9 | frequent | Anterolateral PM | RB | inferior | 168 | 561 | NA*^1^ | NA*^1^ | NA*^2^ |
| NICM10 | trigger | Posterior fascicle | RB | superior | 122 | 407 | normal | normal | yes |
| NICM11 | trigger | LV summit | RB | inferior | 174 | 294 | normal | normal | no |

Abbreviation: ICM, ischemic cardiomyopathy; LV, left ventricle; NICM, non-ischemic cardiomyopathy; PM, papillary muscles.

*^1^: Not assessed because the substrate mapping was not available.

*^2^: Not assessed because the substrate map during the sinus rhythm was not available.

**eTable 2. Comparison between non-ischemic cardiomyopathy patients with and without ventricular fibrillation**

|  | **NICM with VF N=28** | **NICM without VF N=28** | ***P* value** |
| --- | --- | --- | --- |
| Age, years old | 57.5 ± 15.0 | 59.0 ± 13.7 | 0.70 |
| Male | 25 (89.3) | 25 (89.3) | 1.00 |
| LVEF, % | 45.5 ± 13.7 | 45.4 ± 12.7 | 0.98 |
| LV voltage map | 26 (92.9) | 26 (92.9) | 1.00 |
| - Total mapping points | 2342 ± 1208 | 1830 ± 902 | 0.09 |
| - Bipolar LVZ | 16 (61.5) | 16 (61.6) | 0.34 |
| - Unipolar LVZ | 21 (80.1) | 21 (80.1) | 1.00 |
| RV voltage map | 10 (35.7) | 10 (35.0) | 0.78 |
| - Total mapping points | 1521 ± 982 | 2017 ± 971 | 0.30 |
| - Bipolar scar | 3 (30.0) | 3 (30.0) | 0.34 |
| - Unipolar scar | 5 (50.0) | 5 (50.0) | 0.37 |

Data are reported as mean ± standard deviation or n (%).

*Abbreviations: LV, left ventricle; LVEF, left ventricular ejection fraction; LVZ, low voltage zone; RV, right ventricle; VF, ventricular fibrillation*

**Figures**

**eFigure 1. Distal Purkinje network and abnormal Purkinje potential in an electro-anatomical map.**

All the surface points acquired by multi-electrode catheter were reviewed, and the bundle potentials, distal Purkinje networks, and abnormal Purkinje potentials were manually marked. The sweep speed was 200 mm/sec.

**eFigure2.** Sensitivity analysis: comparison of the left ventricular endocardial substrate characteristics between ischemic cardiomyopathy and idiopathic non-ischemic cardiomyopathy

A-C. Ischemic cardiomyopathy (ICM) was associated with larger bipolar low voltage zone (LVZ), bipolar dense scar, and unipolar LVZ.

D-F. The proportion of the bipolar dense scar to the bipolar LVZ, the ratio between unipolar LVZ and bipolar LVZ, and the prevalence of abnormal Purkinje potentials were comparable.

*Abbreviations: ICM, ischemic cardiomyopathy; LVZ, low voltage zone; NICM, non-ischemic cardiomyopathy*

**eFigure 3. Flow diagram of ablation strategy for ischemic cardiomyopathy**

If triggered or clinically frequent premature ventricular complexes (PVCs) were present, the PVCs were targeted for ablation. Inducible ventricular tachycardia was also ablated. Further scar homogenization was performed by the operator’s discrete.

*Abbreviations: VF, ventricular fibrillation; VT, ventricular tachycardia*

**eFigure 4. Flow diagram of ablation strategy for non-ischemic cardiomyopathy**

If triggered or clinically frequent premature ventricular complexes (PVCs) were present, the PVCs were targeted for ablation. Inducible ventricular tachycardia was also ablated. Further scar homogenization was performed by the operator’s discrete.

*Abbreviations: VF, ventricular fibrillation; VT, ventricular tachycardia*

**eFigure 5. Event-free survival analysis for the ventricular arrhythmia recurrence in ischemic cardiomyopathy and non-ischemic cardiomyopathy**

The cumulative incidence of ventricular arrhythmia recurrence was comparable between ischemic cardiomyopathy and non-ischemic cardiomyopathy.

*Abbreviations: CI, confidence intervals; HR, hazard ratio; ICM, ischemic cardiomyopathy; NICM, non-ischemic cardiomyopathy; VA, ventricular arrhythmia*

**eFigure 6. Sensitivity analysis: event-free survival analysis for the ventricular fibrillation recurrence in ischemic cardiomyopathy and idiopathic non-ischemic cardiomyopathy**

The cumulative incidence of ventricular fibrillation recurrence was comparable between ischemic cardiomyopathy and idiopathic non-ischemic cardiomyopathy.

*Abbreviations: CI, confidence intervals; HR, hazard ratio; ICM, ischemic cardiomyopathy; iNICM, idiopathic non-ischemic cardiomyopathy; VF, ventricular fibrillation*

**eFigure 7. Event-free survival analysis for the ventricular fibrillation recurrence in patients with and without premature ventricular complex ablation.**

The cumulative incidence of ventricular fibrillation recurrence was similar between patients with or without premature ventricular complexes ablation.

*Abbreviations: CI, confidence intervals; HR, hazard ratio; PVC, premature ventricular complex.*

**eFigure 8. Comparison of the substrate characteristics between non-ischemic cardiomyopathy with and without ventricular fibrillation**

The bipolar low voltage zone (LVZ), bipolar dense scar, unipolar LVZ, the proportion of bipolar dense scar to the bipolar LVZ, the ratio between unipolar LVZ and bipolar LVZ, and the prevalence of abnormal Purkinje potentials were comparable between non-ischemic cardiomyopathy with and without ventricular fibrillation.

*Abbreviations: LVZ, low voltage zone; NICM, non-ischemic cardiomyopathy*
